# Enhancing patient care: the power of librarian-mediated literature reviews

**DOI:** 10.5195/jmla.2026.2246

**Published:** 2026-04-01

**Authors:** Heather J. Martin, Carrie Grinstead, Danielle Linden

**Affiliations:** 1 heather.martin@providence.org, Providence; 2 carrie.grinstead@providence.org, Providence; 3 danielle.linden@stjoe.org, Providence

**Keywords:** Hospital libraries, Surveys & Questionnaires, Information Services, Expert Searching, Clinical Support, Program Evaluation

## Abstract

**Background:**

Our health system library fields thousands of requests for literature searches each year in support of research, policy, evidence-based practice projects, and care for individual patients. With fewer library staff than comparable institutions and an engaged, multidisciplinary clinical workforce, we face ongoing pressures to do more with less and to demonstrate our value.

**Case Presentation:**

A 2021 article in the *Journal of Hospital Librarianship* offered an existing survey and basic project design that we used to assess our impacts. We adapted, with permission, the survey and methods of “Analysis of a Hospital Librarian Mediated Literature Search Service at a Regional Health Service in Australia,” a quality improvement project authored by Siemensma et al. (2021) [1]. Throughout 2023 we sent the adapted survey to all employees and affiliated clinicians who requested literature searches. The survey included five multiple choice questions as well as a free text box for comments. Respondents were asked to provide simple demographic information and consider the impact and quality of results they received from the librarian.

**Conclusions:**

Our survey-based evaluation of our literature search service underscores the importance of librarian-mediated literature searches for clinical practice, policy development, and patient care. Demonstrating hospital library impacts is increasingly important and increasingly challenging for understaffed teams. Assessments using previously published surveys are feasible for non-academic libraries and serve as compelling cases for the continued and expanded integration of library resources into clinical practice and decision-making.

## INTRODUCTION

Providence is a not-for-profit Catholic health system serving 51 hospitals, 1000 clinics and a comprehensive range of health and social services across 7 US states. With over 122,000 employees including 34,000 physicians, Providence is the 6th largest not-for-profit health system in the United States [2]. The clinical information needs of the organization are supported by the Providence Library. With 9 FTE librarians and 2 FTE library staff, the Providence Library operates at less than 50% of the MLA recommended staffing for Bronze-level service [3,4].

As most of the primary clientele of Providence Library are in clinical positions (e.g., physician, nurse, or pharmacist), mediated literature searching to support patient care is one of our most heavily used services. As a small library team serving a large health system, we need to carefully evaluate the effects of our work, share our successes, and gather information to help us improve. However, for librarians working alone or on understaffed hospital teams, the task of developing and validating new survey tools for evaluating services can be intimidating and time-consuming. Our team identified the survey published by Siemensma et al. (2021) as meeting our needs and integrating well into our team’s existing workflows and tools [1]. We hoped that by adapting and implementing an existing survey instrument, collecting program evaluation data would require only minimal additional staff time and would not affect our day-to-day literature search, article retrieval, collection management, website maintenance, and education and outreach services.

## CASE PRESENTATION

### Methods

We adapted, with permission, the survey and methods of “Analysis of a Hospital Librarian Mediated Literature Search Service at a Regional Health Service in Australia” [1]. We built the survey using REDCap, as it was readily available to the team and we had experience using the tool to collect service statistics [12,13]. We retained Siemensma et al.’s[1] survey questions regarding impacts of and satisfaction with literature search results but adjusted demographic questions for our setting. In late 2022, we submitted our project to our institution’s Institutional Review Board (IRB), who determined that it was not human subjects research and did not require IRB review. Data collection began in January 2023 and ended in January 2024. We sent the adapted survey to all employees and affiliated clinicians who requested literature searches. See Appendix A for the survey. When a librarian completed a search and sent results to the requestor by email, they noted the requestor’s email address in a REDCap form. Each Friday we ran a report of all email addresses that had received literature searches two weeks prior. This time frame was chosen to remain true to the study being reproduced, and in the hope that it would give the requestor enough time to review results and determine quality and impact, while not waiting so long that they may forget specifics.

Like the Australian study, we used a generic email address to send the survey request to our users, as associating the request with an individual member of our staff could result in perceived pressure to respond [1]. We did not want our patrons to feel that their decision to respond or not, or anything they might say in the survey, could affect their future use of our services or their relationships with the library team. Email addresses were noted separately from the survey, used only to distribute the survey link, and not retained. The same link was sent to all participants with no ability to connect a response to an individual, and the survey questions included no identifying information. Participation was voluntary and we did not compensate respondents in any way.

Because the purpose and results of each search were different and may have had different impacts on clinical care or practice, requestors received an email with the survey link each time they requested a new search. However, if one patron requested multiple searches in one week, they would receive only a single survey for that week. We had some concern that frequent requestors would be bothered by receiving many surveys over the course of the year, but we did not receive any negative feedback.

The survey email included introductory language about the project with instructions and a link. The survey included five multiple choice questions as well as a free text box for comments. Respondents were asked to provide demographic information and consider their specific literature search question and the impact and quality of results they received.

We used Excel to compile summary statistics, documenting survey response rate, respondents’ primary job roles, impact on practice, perceived quality of the literature review, and time saved.

### Results

Data collection closed in January 2024 after twelve full months.

We sent 1,048 surveys and received 237 responses, for a response rate of 22.61%. Since we did not collect personally identifying data in the survey, we do not know if each response came from a different patron, if some frequent users of our service responded to the survey more than once throughout the course of the year, or if responses from frequent users were different from those who responded only once.

Respondents were asked to self-identify with the role they felt best described their primary responsibilities within the health system (see Table 1). The most frequently selected roles were nursing staff (65 responses; 27.4%), medical staff (43 responses; 18.1%), administration (36 responses; 15.2%) and educator (35 responses; 14.8%). Respondents were allowed to select more than one role, such as nurse and educator. The survey’s categories were intentionally broad to encompass the range of roles in our complex organization.

**Table 1:** Self-identified roles from survey respondents. Proportions sum to >100% since some respondents selected more than one role.

Role	Number of responses	Proportion of responses
nursing staff	65	27.4%
other	54	22.8%
medical staff	43	18.1%
administration	36	15.2%
educator	35	14.8%
allied health	27	11.4%
researcher	17	7.2%
resident/fellow	4	3.4%
pharmacy	2	0.84%
student	1	0.42%

**Figure 1:**
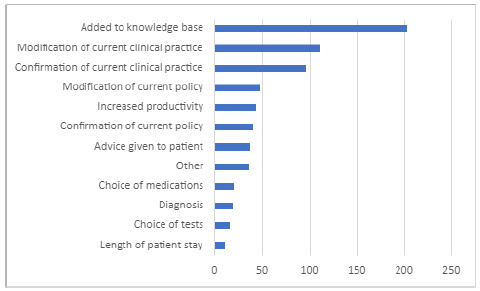
Literature search impacts identified by survey respondents. Respondents were able to select as many impacts as applied to each service interaction.

As with role, respondents were able to select as many impacts as applied for each service interaction (see Figure 1). Respondents selected a median of two impacts, and 29 respondents did not select any. All respondents confirmed that utilizing the librarian-mediated search service saved them time. Thirty-five percent (83) of respondents said our search saved them one to three hours, 29% (68) said four to six hours and 31% (72) said we saved them more than seven hours of their own time. Over 98% (229) of survey respondents rated their search results as either high or very high quality. Eighty-five percent (198) perceived their results as being of very high quality, with 13% (31) rating the results they received as high quality. No respondents rated the search results they received as low quality and only 1.7% (4) rated their results as neither high nor low quality.

We also received 170 comments in the free-text space provided at the end of the survey. Though formal qualitative analysis was not conducted on the free-text comments, we identified some common sentiments within participant responses. Respondents frequently expressed gratitude (60 comments) and positive experiences with a library team member (73 comments). Many also commented on the fast response and the time saved by the service (15 comments). Others described their search results as accurate, thorough, or relevant (39 comments). A sample of representative respondent comments are presented below:

The Providence Librarian has been a huge help to me in the past few years. This year I finally started doing research and although I am the Principal Investigator for this project, I still feel lost at times with how much information is out there. The Librarian is magic and helps me SO much. I sometimes feel like I am over-using them but I truly am so grateful for this service.

[The librarian] was beyond amazing. She produced the perfect literature review in an incredibly short time. I never would have been able to replicate that on my own. What she provided allowed us make a clinical decision that is currently different from our policy. Thank you!

I cannot express how much benefit to clinic productivity, and up to date EBR pt care this program has for our cancer clinics. I do not have the time nor expertise to search the same methods the librarians provide to me. The depth of academic research is top notch and dependable. What a huge mistake to patient care if this resource were to be lost.

The medical librarian services are high quality and the quickest and most comprehensive I have worked with in my career so far. The response time is incredible as is receiving the information needed is exactly what I look for.

Wonderful resource. The search was helpful for prepping of a rare/complex patient and periprocedural/perioperative management. There was a scarcity of literature detailing some specific requests but the thorough search and summary of peer-reviewed and other sources was very much appreciated.

## DISCUSSION

The adoption of an existing tool made this project manageable under time and staffing constraints and allowed us to more easily leverage results for external dissemination and for advocacy within our organization. The approach also builds on an existing body of survey-based assessments from similar settings [1,5–11]. Previous studies document the importance of impacts of information provided by libraries on patient care decisions [1,5]. Like the Australian project that inspired our work, all studies reviewed used surveys, and a few included follow-up interviews [5–10,14–16].

Marshall et al’s landmark 1992 study evaluated hospital library services based on clinical impacts, not simply user satisfaction [5]. In the decades since, Marshall and colleagues have developed larger-scale studies [6,7], and hospital libraries have assessed impacts on patient care decisions, clinicians’ professional development, and return on investment of library services [8,14–16]. Similarly, our survey showed that literature searches added to users’ knowledge base; affected diagnosis, therapy, or the advice given to a patient; modified or confirmed clinical practice; and improved productivity.

A 2014 Marshall study referenced the challenges in accessing patient records and the complexity of clinical decision-making; studies in this area cannot directly attribute clinical outcomes to library resources or services and instead rely on self-report from library users [7]. In 2022, a study based on interviews with medical librarians highlighted a need for new measurements of library impacts [17]. We could not measure effects on patient outcomes, but our results add to the body of literature indicating that evidence compiled by professional librarians affects care decisions.

Impacts may be indirect, since search services add to users’ knowledge base; however, studies also indicate that libraries contribute to a broader culture of inquiry. Brian et al. found that clinicians asked more questions when a librarian was present on rounds, and 45% (154) of respondents to a survey by Brettle at all “reported using the information [provided by the librarian] to deliver training and education to other staff” [10,16]. Our free-text comments suggest that our users also share literature search results with larger teams.

Existing literature consistently demonstrates that search services save time for clinicians [7–9]. Over 50% (140) of our respondents stated that our searches saved them over four hours. A 2022 UK study demonstrated a positive return on investment from a hospital library [8]. While our findings suggest that our services could save organizations money or provide additional value, further evidence is needed to support this claim.

Over 98% (229) of respondents rated the results they received as high or very high quality, similar to the 98.21% satisfaction rate achieved by our Australian colleagues [1]. This highlights our librarians’ professionalism and the trust we’ve earned in our organization.

Finally, our survey results helped to build an overall health system library value case, demonstrating the essential role of library professionals in supporting clinical decision-making and improving patient care. These findings underscored the importance of library staffing even amid budget reduction targets and were recently used to advocate for sustained investment in library personnel. Through a one-sheet flyer and a summary in our widely shared Providence Library Annual Report [19], results reached leadership, frontline clinicians, and key library stakeholders and champions, such as nursing research councils. We presented this work externally at the Medical Library Association 2024 Annual Meeting in Portland, Oregon [20], as well as in internal conferences [21].

Further research may seek improved methods of assessing patient care impacts and explore questions related to library services and clinician well-being and job satisfaction. However, straightforward survey assessments are feasible for small, non-academic library teams and serve as compelling cases for the continued and expanded integration of library resources into clinical practice and decision-making.

Limitations include the possibility of voluntary response bias, as individuals who feel strongly positive or negative may be more likely to fill out the survey. Additionally, no formal qualitative analysis was done and future studies may want to build that into the work plan. Finally, while this study made clear the positive impact that librarian-mediated literature searches had on our clinicians, it is not generalizable beyond our institution. However, we encourage other hospital libraries carry out similar projects.

## Data Availability

Data associated with this article is available from the authors upon request.

## SUPPLEMENTAL FILES

**Appendix A:** Survey Email
