# Supplementary material for: Enhancing patient care: the power of librarian-mediated literature reviews
Source: J Med Libr Assoc. 2026 Apr 13;114(2):164–8. doi: 10.5195/jmla.2026.2246 (PMC13075576; doi:10.5195/jmla.2026.2246)
Supplement: Supplementary file 1 — Appendix A: Survey Email [file jmla-114-2-164-s01.docx]

**Appendix A**

**Survey Email**


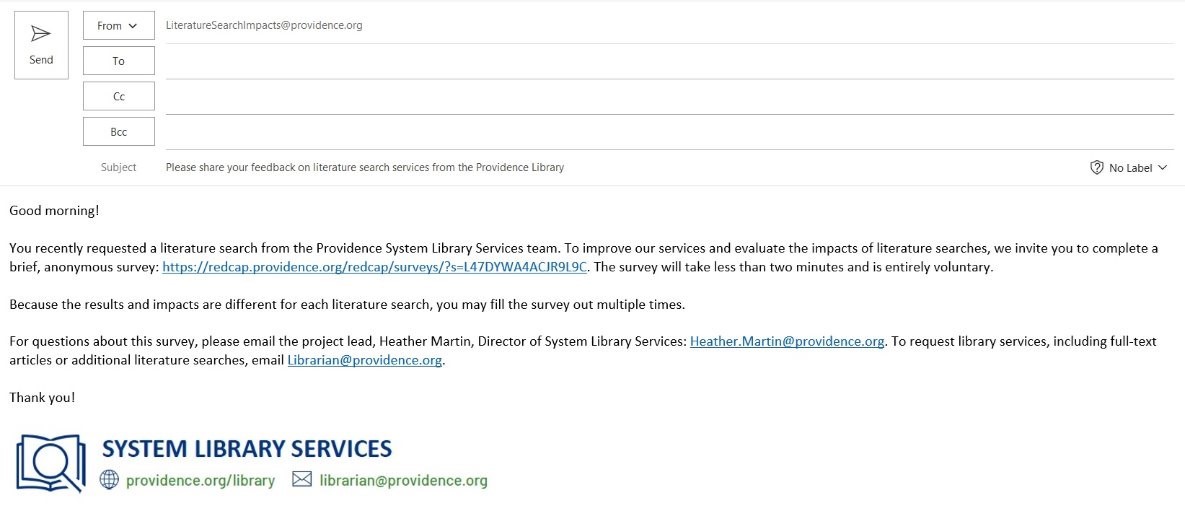
**RedCap Survey Questions**

**Where do you work?** ​

- Alaska
- California
- Montana
- New Mexico
- Oregon
- Texas
- Washington
- Another state
- Outside the US

**What is your primary role? Check all that apply.**​

- Nursing staff
- Allied health
- Administration
- Student
- Researcher
- Medical staff
- Pharmacy
- Educator
- Resident/Fellow
- Other​

**Did the results of this literature search have an impact on any of the following? Check all that apply.**​

- Added to knowledge base
- Modification of current clinical practice
- Confirmation of current clinical practice
- Modification of current policy
- Confirmation of current policy
- Diagnosis
- Length of patient stay
- Choice of tests
- Choice of medications
- Advice given to patient
- Increased productivity
- Other​

**Did receiving these search results save you time?**​

- No
- Saved less than 1 hour
- Saved between 1 and 3 hours
- Saved between 4 and 6 hours
- Saved more than 7 hours​

**How would you rate the quality of the search results?**​

- Very high quality
- High quality
- Neither high nor low quality
- Low quality
- Very low quality​

**Do you have any comments about this search or other library services?**​
